# Supplementary material for: Clinical and Functional Characterization of the Recurrent TUBA1A p.(Arg2His) Mutation
Source: Brain Sci. 2018 Aug 7;8(8):145. doi: 10.3390/brainsci8080145 (PMC6119949; doi:10.3390/brainsci8080145)
Supplement: Supplementary file 1 [file brainsci-08-00145-s001.zip › Supplementary File 1.pdf]

Clinical and functional characterization of the recurrent TUBA1A p.(Arg2His) mutation.

Jennifer F. Gardner, Thomas D. Cushion, Georgios Niotakis, Heather E. Olson, P. Ellen Grant, Richard H. Scott, Neil Stoodley, Julie S. Cohen, Sakkubai Naidu, Tania Attie-Bitach, Maryse Bonnières, Lucile Boutaud, Férechté Encha-Razavi, Sheila M. Palmer-Smith, Hood Mugalaasi, Jonathan G. L. Mullins, Daniela T. Pilz and Andrew E. Fry

Supplementary File 1

Contents **Page**

Figure S1	Sequences from orthologs and paralogs of TUBA1A demonstrating conservation of the Arg2 residue.	2
Figure S2	*In silico* RNA splicing prediction reports.	3
Table S1	*In silico* predictions and population data.	4
Case reports	Detailed clinical descriptions of the four patients with the TUBA1A p.(Arg2His) mutation.	5

Species	Gene	Protein
Mutation	*TUBA1A*	1-MHECISIHVGQAGVQIGNAC-20
H. sapiens	*TUBA1A*	MRECISIHVGQAGVQIGNAC
P. troglodytes	*TUBA1A*	MRECISIHVGQAGVQIGNAC
B. taurus	*TUBA1A*	MRECISIHVGQAGVQIGNAC
M. musculus	*Tuba1a*	MRECISIHVGQAGVQIGNAC
R. norvegicus	*Tuba1a*	MRECISIHVGQAGVQIGNAC
G. gallus	*TUBA1A*	MRECISIHVGQAGVQIGNAC
X. tropicalis	*tuba1b*	MRECISIHVGQAGVQIGNAC
D. rerio	*tuba1a*	MRECISIHVGQAGVQIGNAC
S. cerevisiae	*TUB3*	MREVISINVGQAGCQIGNAC
P. vanneervenii	*btubB*	MREILSIHVGQCGNQIADRF
H. sapiens	*TUBA1B*	MRECISIHVGQAGVQIGNAC
H. sapiens	*TUBA1C*	MRECISIHVGQAGVQIGNAC
H. sapiens	*TUBA3C*	MRECISIHVGQAGVQIGNAC
H. sapiens	*TUBA3D*	MRECISIHVGQAGVQIGNAC
H. sapiens	*TUBA3E*	MRECISIHVGQAGVQIGNAC
H. sapiens	*TUBA4A*	MRECISVHVGQAGVQMGNAC
H. sapiens	*TUBA8*	MRECISVHVGQAGVQIGNAC
H. sapiens	*TUBB*	MREIVHIQAGQCGNQIGAKF
H. sapiens	*TUBB1*	MREIVHIQIGQCGNQIGAKF
H. sapiens	*TUBB2A*	MREIVHIQAGQCGNQIGAKF
H. sapiens	*TUBB2B*	MREIVHIQAGQCGNQIGAKF
H. sapiens	*TUBB3*	MREIVHIQAGQCGNQIGAKF
H. sapiens	*TUBB4A*	MREIVHLQAGQCGNQIGAKF
H. sapiens	*TUBB4B*	MREIVHLQAGQCGNQIGAKF
H. sapiens	*TUBB6*	MREIVHIQAGQCGNQIGTKF

Figure S1. Sequences from orthologs and paralogs of TUBA1A demonstrating conservation of the Arg2 residue. Alignments of the first 20 amino acids of each protein derived from Ensembl comparative genomics resources (www.ensembl.org). The mutated residue is underlined and in red. The Arg2 residue of TUBA1A is highly conserved across species and tubulin isoforms, including the bacterial tubulin btubB found in *Prosthecobacter vanneervenii*.

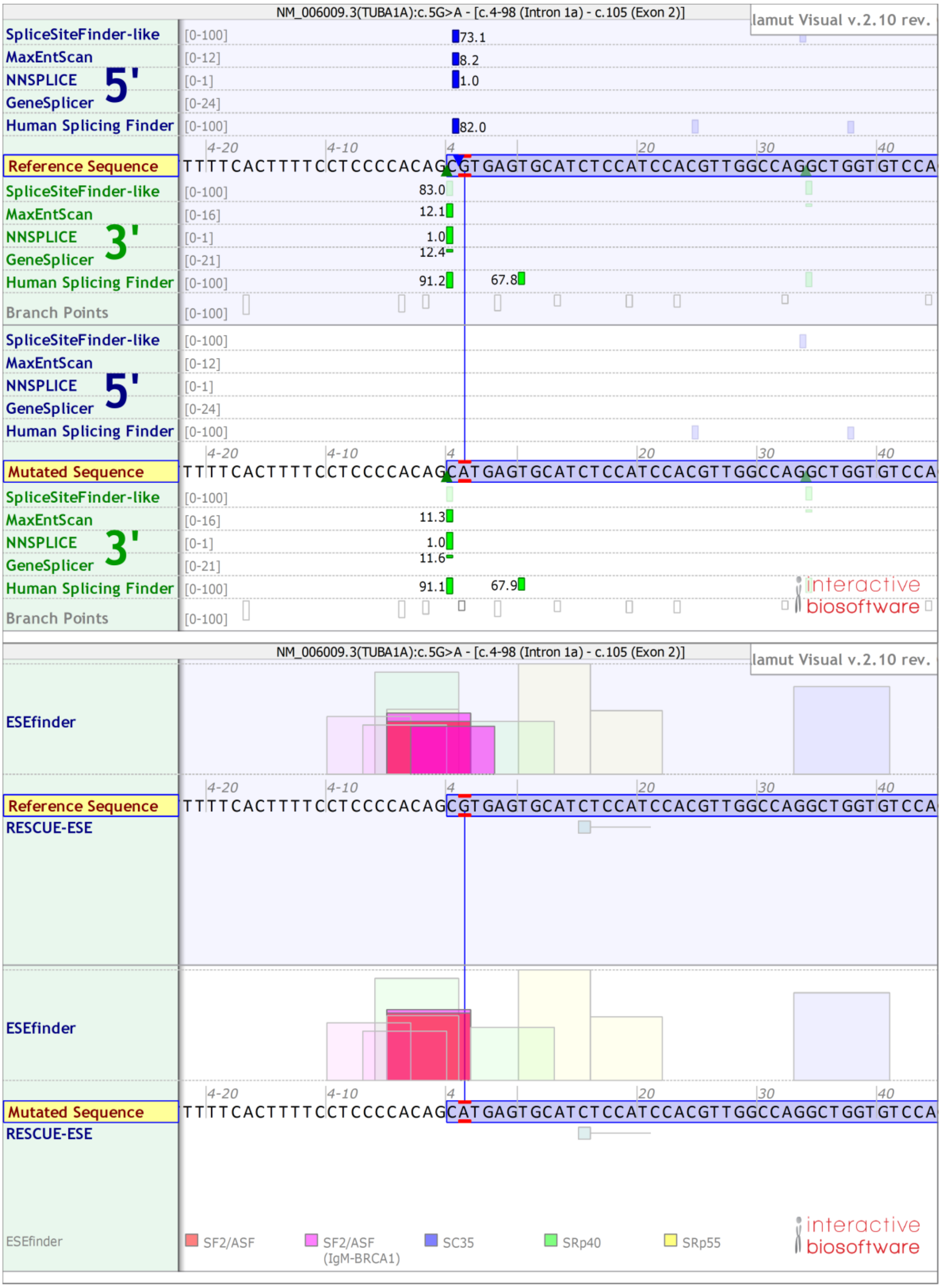

Figure S2. *In silico* RNA splicing prediction reports. Screenshots from the Alamut Software Suite assessing the impact of the c.5G>A mutation on splicing at the adjacent splice acceptor site (3’). The splicing prediction tools are consistent in suggesting limited effects on the splicing at this site.

Table S2. ***In silico*** **predictions and population data.**

**Variant information**	
**Genomic coordinate (GRCh37/hg19)**	chr12:g.49580615C>T
**cDNA**	NM_006009.3: c.5G>A
**Protein**	NP_006000.2: p.(Arg2His)
**dbSNP number**	rs587784491
In Silico **predictions**	
**PhyloP**	2.639
**Grantham**	29
**SIFT**	Deleterious (0.009)
**Polyphen 2**	Possibly Damaging (0.814)
**MutationTaster**	Disease Causing (1)
**CADD (PHRED-like)**	Top 0.1% (34)
**M-CAP**	Possibly Pathogenic (0.245)
**Population data**	
**ExAC**	No entry
**gnomAD**	No entry

Case reports

Patient 1

This male patient was the second child of non-consanguineous White European/South East Asian parents. He had one older sister who was well. There was no significant family history. Chorionic villus sampling was performed due to maternal age and showed a normal male karyotype. There were concerns at 20 weeks gestation because growth parameters (including head size) were generally small (his mother and sister were known to have had low birth weight). Regular growth scans were performed. He was born at 39 weeks gestation by normal vaginal delivery. His birth weight was 2250 g (-2.5 SD). Birth head occipitofrontal circumference (OFC) was 30 cm (-3.6 SD). At six weeks of age his head circumference was 34 cm (-3.7 SD). He began smiling socially around 8 weeks. Concerns were raised at this stage because of his feeding difficulties and poor weight gain. His weight gain improved with nasgoastric feeding but the microcephaly persisted. Videofluoroscopy showed a safe but hesitant swallow. The patient sat at 1 year of age.

At 28 months of age the patient had moderate global developmental delay. He could sit and crawl, but he was not yet walking. His speech consisted of multi-syllable babbling. He had excess dribbling. The patient had no seizures at this point. There were no concerns about his vision or hearing. Oral intake continued to be poor and he was mainly gastrostomy fed. On examination he had a mild metopic ridge, prominent ears, epicanthic folds and hypotonia. His weight was 10.7 Kg (-2.0 SD) and OFC 42.5 cm (-6.1 SD). The patient had an episode of focal onset status epilepticus at 3 years of age, and subclinical left occipital lobe seizures on EEG just following this event. He then remained mostly seizure free on oxcarbazepine apart from one breakthrough seizure (6 minutes, hemiclonic) at 4 years in the setting of

hyponatremia. The hyponatremia was likely related to his oxcarbazepine treatment. The patient was last reviewed at 4 years of age. His weight was 14.9 kg (-0.9 SD), height 96.8 cm (-1.4 SD), and OFC 45 cm (-4.9 SD). At this point he was starting to stack blocks, had a few signs and was able to point at pictures but was not really making choices well yet. He was walking with an unsteady gait. On examination, he had a very mild right hemiparesis.

Array CGH and extensive metabolic investigation (including very long chain fatty acids, serum amino acids, urine organic acids and creatine kinase) were all normal. An MRI brain scan at 3 years of age revealed bilateral perisylvian polymicrogyria. The basal ganglia were dysmorphic (globular with incomplete formation of the anterior limb internal capsule). There was mild hypoplasia and dysplasia of the cerebellar vermis, and a small pons. A targeted next-generation sequencing gene panel reported a mutation, c.5G>A, in exon 2 of *TUBA1A*. This resulted in a missense change, p.(Arg2His). Parental testing demonstrated the mutation was *de novo*.

<u>Patient 2</u>

This male patient was the first child of non-consanguineous Greek parents. There was no significant family history. The pregnancy with the patient was uncomplicated. Antenatal scans were normal. He was born by normal vaginal delivery at term. At birth his weight was 3740 g (+0.4 SD), length 50 cm (-0.5 SD) and OFC 34 cm (-0.9 SD). The patient fed well in the postnatal period. Concerns were raised at 6 months of age due to poor head growth. At 7 months of age he was noted to be unable to fix and follow, had no social smile, and was hypotonic. The patient developed seizures at 12 months.

The patient was last reviewed at 32 months of age. He had severe global developmental delay. He sat at 14 months, but was not yet walking or talking. He had poor social interactions and no speech. He took pureed foods orally. He continued to have daily seizures (absence and generalised tonic-clonic). On examination, he had microcephaly (OFC 43 cm, -5.9 SD), profound hypotonia, and no visual attention. His height was 95cm (+0.7 SD) and weight 13 kg (-0.6 SD). He was not dysmorphic. There were no concerns about hearing. ABSR were normal. Visual evoked potentials and electroretinogram were normal. EEG showed multifocal spikes with slow background activity. Extensive metabolic investigation and TORCH screen were normal. An MRI brain scan (performed at 6 months of age) showed small brain volume with dysmorphic basal ganglia and thin corpus callosum. The pons was small. There was dysplasia and hypoplasia of the cerebellar vermis. No cortical malformation was observed. A targeted next-generation sequencing gene panel found a c.5G>A, p.(Arg2His) mutation in *TUBA1A*. Parental testing demonstrated the mutation was *de novo*.

Patient 3

This male patient was the first child of non-consanguineous parents of Indian heritage. There was no significant family history. The pregnancy with the patient was uncomplicated. Antenatal scans were normal. He was delivered at term by normal vaginal delivery. Birth weight was 2778 g (-1.6 SD). Birth head circumference was 33 cm (-1.7 SD). The patient was well in the perinatal period. Concerns were raised at 7 months due to his delayed developmental milestones, hypotonia and poor head growth. The patient developed hand-flapping at 10-12 months and sat unsupported at 14 months.

The patient was last reviewed at the age of 37 months of age. He had global developmental delay. He was unable to walk and could only say a few single words. He was able to eat soft

foods orally, but he had difficulty chewing and had poor weight gain. He had no history of seizures and EEG at 2 years was normal. On examination, he was microcephalic (OFC 45cm, -4.6 SD). His height was 93.5 cm (-0.7 SD) and weight 11.8 kg (-2.1 SD). The patient had some minor physical anomalies including small testes, wide-spaced nipples and 4 cafe au lait marks. TORCH screen and metabolic investigations were normal. The results of array CGH and a nine gene X-linked intellectual disability panel were normal. MRI brain (at 19 months of age) showed a thin, but complete, corpus callosum and dysmorphic, prominent basal ganglia without clear differentiation between putamen and head of the caudate. The cortex appeared normal with no polymicrogyria or any other cortical dysplasia. There was dysplasia and hypoplasia of the cerebellar vermis. Trio-based whole-exome sequencing identified a *de novo* mutation c.5G>A, p.(Arg2His) mutation in *TUBA1A*. The mutation was confirmed by Sanger sequencing. Parental testing demonstrated the mutation was *de novo*.

Patient 4

This was a male fetus of a healthy Caucasian couple with unremarkable family history. The pregnancy was terminated at 36 weeks gestation for brain anomalies including cerebellar hypoplasia and abnormal appearance of the corpus callosum. Post-mortem examination identified some dysmorphic features including a narrow forehead, epicanthic folds, retrognathia, large ears and long digits. All growth parameters were within normal range with a head circumference of 25th centile (-0.6 SD). There was a single umbilical artery and small accessory spleen. Macroscopic examination of the brain showed reduced brain weight (5th centile, -1.6 SD) and short corpus callosum with absent rostrum. Neuropathology examination found bilateral perisylvian polymicrogyria. At the supratentorial level, callosal fibers and corticospinal tracts (CST) were hypoplastic. The brainstem was shortened and dysmorphic, displaying a Z-shaped kink. At the level of the cerebral peduncles, the CST were

present but reduced in size. The pons was reduced in size in its basilar part. In the pons the CST were present at the junction with the peduncles but showed a chaotic pattern in between the pontine nuclei. The transverse pontine fibers were also reduced, and associated with cerebellar heterotopias and hypoplastic deep nuclei. At the level of the medulla, the pyramids were present but hypoplastic. The inferior olivary nuclei were also reduced in size. Neuronal heterotopia of the olivary nuclei were noted. At the cervical spinal cord level, crossing CST were absent. Cerebellar foliation was normal, but lamination was impaired with rare and misaligned Purkinje. Next generation sequencing of a panel of genes associated with abnormalities of the corpus callosum identified a *de novo* mutation in *TUBA1A*, c.5G>A, p.(Arg2His).
